# The Relationship of Trunk Muscle Activation and Core Stability: A Biomechanical Analysis of Pilates-Based Stabilization Exercise

**DOI:** 10.3390/ijerph182312804

**Published:** 2021-12-04

**Authors:** Kyeongjin Lee

**Affiliations:** Department of Physical Therapy, College of Health Science, Kyungdong University, Wonju 24764, Korea; kjlee@kduniv.ac.kr

**Keywords:** electromyography, Pilates based exercises, kinematics, internal oblique muscle, multifidus

## Abstract

Pilates is an effective exercise method for rehabilitating musculoskeletal disorders as its principles are based on the activation of local muscles. This study aimed to compare the subjects with and without Pilates experience to find out the effect of the experience on the core muscle activity and muscle co-contraction, and to examine the relationship between the core muscle activation level and the kinematic data. This study involved 32 subjects, including 16 experienced Pilates practitioners and 16 non-experienced subjects. The knee stretch on the reformer was performed in three different positions: flat back with a neutral pelvis, round back with posteriorly tilted pelvis (RPP), and extended back anteriorly tilted pelvis (EAP). The electromyography of the internal oblique (IO), rectus abdominis (RA), multifidus (MU), and iliocostalis lumborum (IL) muscles were measured, as well as kinematic data from a 3D motion analysis system. Compared to the non-experienced subjects, the experienced subjects activated the IO muscles more than the RA muscles, and the most significant difference was seen in the RPP position (*p* < 0.05). The experienced patients activated the MU muscles more often than the IL muscles, with the most significant difference observed in the RPP position and the least significant in the EAP position (*p* < 0.05). All kinematic data and muscle activity (IO, IO/RA ratio, MU/IL ratio) showed significant differences between the experienced and non-experienced subjects (*p* < 0.05). The subjects presented a moderate correlation between muscle activation and core stability. It was confirmed that the experienced Pilates practitioners activated the abdominal and low back core muscles effectively, and the stability of the pelvis and trunk were better than that of the non-experienced participants. In addition, the better the trunk stability was maintained, the larger and more accurate movement of the mobility segment was observed.

## 1. Introduction

For an effective biomechanical movement that minimizes joint loading, proximal stabilization must precede the movement of the distal extremities [1]. Proximal stabilization harmonically and functionally leads to movement of the extremities [2,3]. Spinal muscles that provide proximal stabilization are divided into the global and local muscles. Global muscles, including the erector spinae muscles of the back and the rectus abdominis muscles of the abdomen, are involved in overall stability and mobility by creating power and large movements [4,5]. The multifidus, a local muscle attached directly to the spine, controls precise movement and provides inter-spinal stability [6]. The transverse abdominis and internal obliques are the other local muscles located in the deep layers of the abdomen that provide stability by connecting the pelvis and ribcage to the spinal fascia [7]. The local muscles contract before the global muscles to maintain proximal stability, thus providing support to the actions of the global muscles [2,8,9]. In addition to these muscles, the multifidus, pelvic floor muscles, and diaphragm form a local muscle system in the lower back to provide trunk stability [3,10]. As a feedforward system, the trunk stabilizers contract prior to the movement of the extremities, thus enhancing the stability of the movement [2].

To achieve proximal stabilization, the local muscles must be active while the global muscles are not over-activated. Training methods emphasizing isolated contraction of the local muscles, as well as the need for proximal trunk exercises have been on the rise [11,12]. Based on these principles, proximal trunk exercises, including the use of a pressure biofeedback unit or real-time ultrasound, were adopted [12,13]. All of the exercises included in Pilates underline activation of the local muscles to implement a whole-body rhythmic movement [5].

Pilates effectively activate these local muscle systems and, therefore, are on the same track as the principles mentioned above. Pilates breathing can induce the activation of trunk stabilizers in order to take on the role of arranging the firing patterns of muscle recruitment [14]. An imprinted position is a Pilates posture with a slightly posterior tilted pelvis that activates the abdominal local muscles while maintaining the neutral position of the pelvis [15]. Centralization is one of the basic principles of Pilates, which highlights the active use of the trunk local muscles and is applied to all Pilates exercises [16]. Hence, Pilates is drawing more attention recently due to its positive effects on whole-body strengthening and its ability to enhance flexibility on the basis of proximal stabilization. Recently, clinical experts have recommended Pilates as a therapeutic exercise approach to rehabilitate disorders of the spine, such as back pain and scoliosis [17,18,19].

A reformer is one of the main apparatuses used in Pilates; it allows for changes in the placement of the carriage using a stopper, and springs that connect the reformer and carriage can set the intensity and amount of range of motion in an exercise. The carrier simultaneously provides movement and resistance. A study proved that exercises on the reformer activate the abdominal core muscles more than when the same exercises are performed on a mat [15].

In a previous study, activation levels of core muscles were high, and co-activations of lumbar and abdominal muscles were more frequent among subjects with Pilates experience than in non-experienced subjects during Pilates exercises [20]. This finding suggests that Pilates can activate muscles that stabilize the trunk [20,21].

The knee stretch is a stabilization exercise using the reformer that involves movement of the lower extremities while maintaining a quadruped position and active stabilization of the trunk and upper extremities on the carriage. During the knee stretch exercise, the core muscles of the back and abdominal contract result in co-contraction of these two muscle groups.

To enhance proximal stability, activation of the global muscles has to decrease while activation of the local muscles increases, resulting in co-contraction with an adequate muscle activity ratio [22,23]. The activity ratio of the local muscles has to be relatively higher than that of the global muscles to adjust co-contraction delicately [8,24]. Thus, it is crucial to evaluate the activation levels of the local muscles, as well as the activity ratio of the local muscles and global muscles. Heretofore, most studies of Pilates have only addressed observations of movement using electromyography (EMG) [16,20], while only a few have investigated the quality of movements using motion analysis.

Therefore, the purpose of this study is to compare the subjects with and without Pilates experience to find out the effect of the experience on the core muscle activity and muscle co-contraction, and to examine the relationship between the core muscle activation level and the kinematic parameter.

## 2. Materials and Methods

### 2.1. Subjects

Professional Pilates practitioners were recruited online, and of the 62 instructors who volunteered, 16 participants who understood and performed the exercise protocols appropriately and met the criteria were selected. Inclusion criteria were healthy females aged between 21 to 35 who had at least one year of Pilates experience. Participants who had disturbances of balance due to orthopedic or neuropathic disorders, cardiopulmonary disorders, surgery within six months, or any other disabilities were excluded. Non-experienced participants were also recruited online, and of the 54 healthy female adults who volunteered, 16 participants who met the inclusion criteria were selected. Inclusion criteria were healthy females aged between 21 to 35 who had not participated in any other exercises in the last 6 months. The exclusion criteria were the same as the professional Pilates practitioners. All 32 participants met the criteria. This study was approved by the Ethics Committee of Kyungdong University, and all experiments were conducted in a Pilates center located in a university in Seoul, Republic of Korea. Before the experiment, the purpose and procedure of the study were explained, and the subjects provided written consent. The computer program G-power 3.19 (Heinrich Heine University Düsseldor, Düsseldorf, Germany) was used to decide sample size, the significant level was set to 0.05, and statistical power was set to 0.8. The effect size was based on the activation of the internal oblique through the pilot test, which is the critical factor of this study. The effect size was calculated to 0.25. The total sample size for the experiment was set to 28, considering a 10% dropout rate of 32.

The general characteristics of the 16 experienced and 16 non-experienced subjects are presented in Table 1. The height, weight, and body mass index values of the two groups were statistically homogeneous. There were no significant differences in the general characteristics.

### 2.2. Pilates-Based Stabilization Exercise

**Figure 1:**
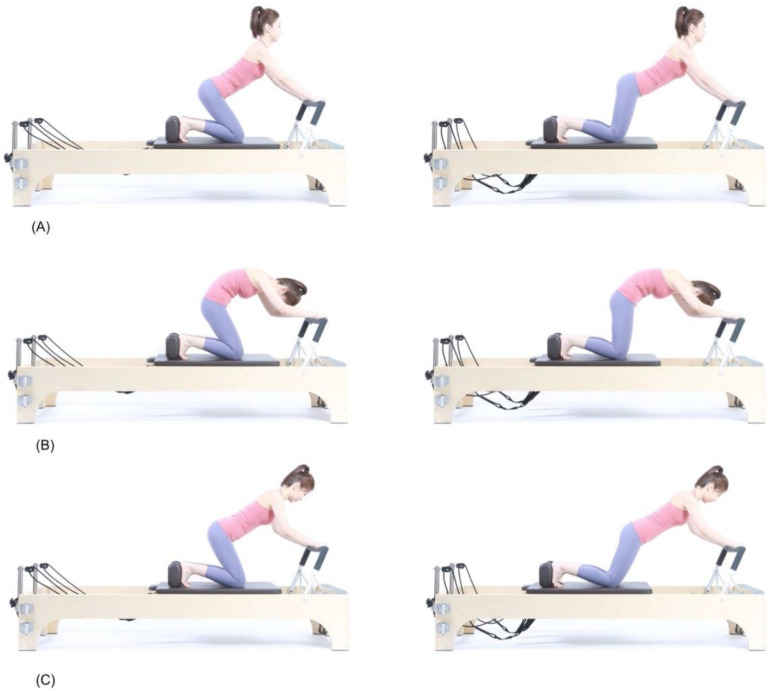
Knee stretch exercise. (**A**) Starting position (left) and end position (right) of the extended back with an anteriorly tilted pelvis (EAP) position. (**B**) Starting position (left) and end position (right) of the round back with a posteriorly tilted pelvis (RPP) position. (**C**) Starting position (left) and end position (right) of the flat back with a neutral pelvis (FNP) position.

Subjects performed knee stretch using a Pilates reformer (V2 Max™ Reformer, Toronto, ON, Canada) as the Pilates-based stabilization exercise. The subjects were instructed to perform knee stretches on a reformer. The tension for springs used were set between 2.4 and 3.3 kg (75% Reformer spring, Merrithew™, Toronto, ON, Canada). The subjects kneeled on a movable carriage with their feet placed against the shoulder rests. Both hands held onto the footbar, and the subjects were instructed to lift their hips moving away from the legs. Knee stretches were performed using three different methods with different starting positions (Figure 1). In the first method, the subjects performed the exercises while maintaining a flat back with a neutral pelvis (FNP). At this point, they were instructed to look 45° downward and maintain their trunk and hip positions. For the second method, they maintained a round back with a posteriorly tilted pelvis (RPP) while performing the movements. Each subject tilted their pelvis posteriorly and flexed their spine to create a round back posture before performing the tasks. They spontaneously looked down toward their knees. In the final method, the subjects were instructed to maintain an extended back with an anteriorly tilted pelvis (EAP) during the knee stretch movements. The subjects looked towards the front and maintained an extended posture throughout the task. For all three methods, the starting position was in the ‘home’ position, which was the point where the carriage was closest to the footbar. The subjects were instructed to move the carriage away from the footbar as far as possible while maintaining the starting position. At the endpoint of the stretch, the subjects maintained their position for one second before bringing the carriage to the home position. Before the measurement, all procedures and cautions were explained to each participant. The subjects practiced the movements three times before the trials. A total of 40 s were required to perform five repetitions, with each repetition comprising 8 s:3 s for the moving part and 1 s for the holding part, with five repetitions performed for each condition.

### 2.3. Data Collection

Surface electromyography (Ultium EMG^®^, Noraxon, AZ, USA) was used to measure muscle activation with dual EMG wet gel electrodes (single electrode T246H, SEEDTECH, Gyeonggi-do, South Korea). All electrodes were placed after shaving and abrading the skin with alcohol. The position of the electrodes was as follows: internal oblique, 2 cm medial and inferior to the anterior superior iliac spine; rectus abdominis, 3 cm from the midpoint line of the umbilicus; iliocostalis lumborum, 2 cm from the spinous process of the lumbar (L1) vertebra; multifidus, 3 cm from the midpoint line from the spinous processes of the L1 to L5 vertebrae. The activation levels of the internal oblique (IO), rectus abdominis (RA), multifidus (MU), and iliocostalis lumborum (IL) muscles were examined. To compare the activation difference between the superficial muscles (IL, RA) and deep muscles (MU, IO), the MU value was divided by the IL value (MU/IL ratio), and the IO value was divided by the RA value (IO/RA ratio) for analysis. The sample rate was 2000 Hz with a band filter of 20–400 Hz and notch filters of 60 Hz. The raw data were processed into root-mean-square (RMS) values with a window of 60 ms after rectification and smoothing.

**Figure 2:**
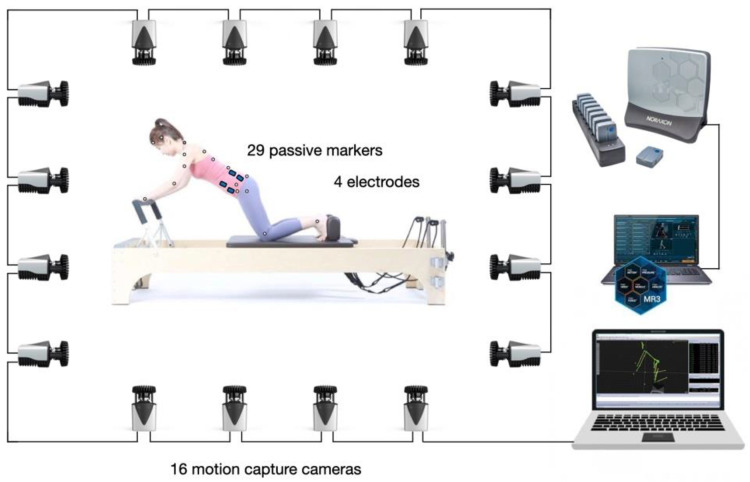
The diagram of 3D motion capture system and electromyography for kinematic data.

To obtain kinematic data, we used 3D motion analysis. The movements of the subjects were captured using 16 infrared cameras (Qualisys Miqus M3, Qualisys AB, Gothenburg, Sweden) at 200 Hz, and the data were obtained and analyzed using the Qualisys motion capture system (Qualisys, Qualisys AB, Gothenburg, Sweden). A total of 29 passive markers were attached to the subjects at the following locations: at the top of the head, above the left and right ears, on the spinous processes of C7, T3, T7, T12, L5, the sacrum, and the left and right acromions; on the lateral epicondyles of the elbows; on the wrists, anterior superior iliac spines (ASIS), iliac crests, posterior superior iliac spines (PSIS), greater trochanters, lateral and medial condyles of the knees; and on the lateral malleolus of the ankles. Four additional markers were placed on the reformer at both ends of the footbar, and both ends of the carriage were placed on the side close to the footbar. Marker coordinates from the motion capture was extracted through Qualisys track manager (Qualisys, Qualisys AB, Gothenburg, Sweden) then analyzed with Matlab (Matlab 2021a, Mathworks Inc., Natick, MA, USA). Signals were processed with a low-pass Butterworth filter of the motion capture software. (Figure 2).

To assess trunk stability, we analyzed the angle of trunk sway. Pelvic stability was calculated from the angle of pelvic sway. To assess trunk stability, we analyzed the angle between the right wrist to the acromion and between the acromion to the spinous process of T12. The range of the angles during the task was collected. Pelvic stability was calculated from the angle between the spinous process of T12 to the sacrum and from the sacrum to the right ASIS. The number of peaks of the changes in angles that occurred during the task was measured. The ranges of the knee stretch angles were measured to assess mobility while performing knee stretches. The angle between the end of the carriage and the lateral epicondyle of the knee to the greater trochanter was measured. The ranges of the angles during the tasks were then analyzed. To define the positions of the hip joint in relation to the knee joint at the starting position, we measured the carriage back angle. From the starting position, the greatest angle between the carriage and lateral epicondyle of the knee and the lateral epicondyle of the knee and the greater trochanter was calculated and subtracted by 90°.

### 2.4. Statistical Analysis

Normal distribution of the data was examined using the Shapiro–Wilcoxon test. The data were expressed as averages with standard deviations. Independent *t*-tests were used to compare the dependent variables between the two groups. Muscle activation among the different groups and conditions was compared using a two-way repeated ANOVA. Post hoc tests were performed by Bonferroni’s test. Pearson’s correlation analysis was performed to analyze the correlation between the kinematic data and muscle activation data. All analyses were performed using SPSS version 23.0 (SPSS Inc., Chicago, IL, USA) for Windows, and a *p* value < 0.05 was considered statistically significant.

## 3. Results

### 3.1. Muscle Activity during Knee Stretch Exercise

Table 2 displays the EMG muscle activation results from the knee stretch exercise. There were significant differences (*p* < 0.001) between the experienced and non-experienced groups in terms of IO after analyzing muscle activity, with the experienced group activating more muscles during the task. Differences between positions or interactions between groups and positions were not significant. There were no significant differences or interactions in terms of RA activation. There was a significant difference in the IO/RA ratio between the groups (*p* < 0.001), with the experienced group activating more IO compared to the RA group. The greatest difference was observed in the RPP position. There was no difference between the positions, but group-position interactions were observed (*p* < 0.05).

There were no significant differences or interactions in terms of MU activation. IL activities only demonstrated inter-position differences (*p* < 0.001), with both groups activating the IL muscles the most in the EAP position and the least in the RPP position. Differences between positions or interactions between groups and positions were not significant. There were significant differences in the MU/IL ratio between the groups (*p* < 0.05) and between the positions (*p* < 0.001). There was also a significant group-position interaction (*p* < 0.05). Overall, the experienced group activated the MU muscles more than the IL muscles while the non-experienced group did not. Both groups showed the highest levels of muscle activation in the RPP position and revealed the smallest MU/IL ratio when testing in the EAP position.

### 3.2. Kinematic Analysis of the Knee Stretch Exercise

The kinematic data from the motion analysis during the knee stretch exercise are presented in Table 3. There were significant differences between the experienced group and the non-experienced group (*p* < 0.05), and between different positions (*p* < 0.05), with the experienced group being able to stabilize the pelvis more than the non-experienced group. However, there was no group-position interaction. The experienced group was able to stabilize the pelvis the most when in the FNP position and the least when in the EAP position, while the non-experienced group was able to stabilize the pelvis the most in the RPP position and the least in the EAP position. There were significant differences between the groups (*p* < 0.05) and between the positions (*p* < 0.05). There was no group–position interaction. The experienced group was able to stabilize the trunk more than the non-experienced group during the tasks. Both groups showed the least stabilization when in the RPP position. For the carriage back angles, there were differences between the groups (*p* < 0.05) and between the positions (*p* < 0.001), but the group interaction was not significant. Both groups brought the carriage back closest to the home position when in the RPP position, while they struggled the most when in the EAP position. There were significant differences between the groups (*p* < 0.05) and between the positions (*p* < 0.001) in terms of knee stretch angles. The group–position interaction was also significant (*p* < 0.05). The experienced group moved in a larger range than the non-experienced group during all tasks.

### 3.3. Correlation between Muscle Activation and Kinematic Data

The correlations between muscle activation and kinematic data are presented in Table 4. There was a significant correlation between pelvic stability and the IO/RA ratio (*r* = 0.419). Carriage back angles and IO activation (*r* = 0.444), as well as carriage back angles and RA activation (*r* = 0.518) also showed significant correlations. Knee stretch angles and IO activation showed a significant correlation (*r* = 0.506). Carriage back angle and activation of RA, and knee stretch angle and activation of IO showed moderate correlation while other variables showed small correlation.

## 4. Discussion

This study investigated the relationship between movement and activation of local muscles by evaluating kinematic parameters using a 3D motion analysis system. Thus, this study compared participants experienced and non-experienced in Pilates while they performed Pilates-based stabilization exercise; this was done to evaluate subjects’ muscle activation of the abdomen and back and analyze their kinematic data. The main findings in this study were significant differences in muscle activity and kinematic data between experienced and non-experienced subjects. All kinematic data (pelvic stability, trunk stability, carriage back angle, and knee stretch angle) showed significant differences between the two groups, and muscle activity (IO, IO/RA ratio, MU/IL ratio) showed significant differences between the experienced and non-experienced subjects.

This study compared muscle activation between subjects experienced and non-experienced in Pilates; the results showed that experienced participants activated the IO muscles more than non-experienced participants. The experienced subjects also showed higher IO/RA and MU/IL ratios. Based on the results, experienced subjects utilize local abdominal and back muscles more efficiently than non-experienced subjects. In the study by Barbosa et al. [20], the Pilates experienced group always had higher TrA/IO activations than their target level; meanwhile, none of the participants in the non-experienced group reached the target level, and they only managed to reach 50% of the experienced group’s achievements. The experienced subjects reached nearly 50% or greater of the maximum voluntary isometric contraction; however, the non-experienced subjects never reached this. Their results showed a strong correlation between Pilates’ experience and muscle recruitment levels. Panhan et al. [25] compared multifidus activation in the same groups using submaximal isometric trunk extension. Their results demonstrated that while there were no differences in activation levels, there were significant differences in isometric peak torque levels, leading them to conclude that the neuromuscular efficiency of the experienced group was significantly higher. However, the actual neuromuscular efficiency could not be confirmed because the authors did not examine the activation of the erector spinae. Moon et al. [26] have compared thickness of deep muscles and activation of surface muscles between Pilates and resistance exercise instructors during four different stabilizing exercises. During the exercises, the transverse abdominis were significantly thicker in the Pilates group compared to the other two groups, and IO were thicker in the Pilates and resistance exercise group compared to the control group. Consequently, they have emphasized the effectiveness of Pilates to increase abdominal deep muscle thickness.

In this study, the experienced group activated the IO muscles more than the RA muscles, and the MU muscles more than the ES muscles. Because the experienced subjects were trained to activate local muscles through breathing techniques and proximal stabilization during movements, they seemed to recruit local muscles and use them as stabilizers more efficiently. Proximal stabilization from the local muscles is essential for accomplishing functional tasks while preventing excessive loads on the vertebrae during movements [7,14]. Movements of the extremities without adequate proximal stabilization can stress the spine and soft tissues, resulting in abnormal functions of balance and posture control, which may increase one’s risk of injury [8,9]. Patients with lower back pain due to spinal instability are known to present with local muscle weakness and functional disabilities [27]. In addition, over-activation of the global muscles can overload the spine and cause pain [2]. Therefore, Pilates can be a reliable way for clinicians to treat patients as they promote local muscle activity over global muscle activity. Yang et al. [18] mentioned in their study that Pilates-based core exercise is an effective therapeutic modality for patients with chronic low back pain that can decrease pain and increase quality of life. Another study that investigated the effect of Pilates on chronic non-specific low back pain confirmed that Pilates decreased pain and increased muscle thickness of the MF and abdominal core muscles [17].

A correct knee stretch exercise means that the lower extremities are mobile while the trunk and upper extremities are stabilized, with ideal spine alignment maintained during alterations of pelvic positions. In this study, muscle activation in three different positions of the pelvis, including FNP, RPP, and EAP positions, were measured. To analyze accurate mobility and stability, a 3D motion capture system was used. From the results of the motion analysis, the experienced subjects stabilized their pelvis to keep it more still during the movements compared to the non-experienced subjects. The experienced subjects accomplished the movements while activating local muscles to maintain trunk stabilization, whereas the non-experienced subjects showed relatively considerable pelvic sway. Pelvic stability was negatively correlated with the IO/RA ratio, indicating that when the IO muscles are more activated than the RA muscles, the motion of the pelvis is small; therefore, it is more stabilized and safer to perform movements. When comparing the three different positions of the pelvis, the RPP position activated the IO muscles the most. In this position, the IO muscles were strongly recruited to maintain trunk flexion. On the other hand, muscle activation was lowest in the EAP position, resulting in the lowest pelvic stability. Our assumption is that trunk extension in the EAP position causes passive insufficiency, such that the muscle cannot adequately contract.

In all three pelvic positions, the experienced subjects presented stronger pelvic and trunk stability than the non-experienced subjects. It seems that the experienced subjects were able to move their legs in a greater range of motion due to their ability to maintain pelvis and trunk stabilization while moving their legs. Good proximal stabilization reflects the ability of the trunk muscles to consciously stabilize the spine and pelvis against an external force [22,27]. Securely controlled local muscles can maintain ideal alignments of the spinal segments, which may eventually decrease redundant activities of the global muscles. In terms of trunk stability, the experienced subjects showed little movement of the trunk during the exercises compared to the non-experienced subjects. Neither group maintained trunk stability while in the RPP position. This may be due to the passive insufficiency of the back muscles when they are elongated in the upper trunk flexed position, ultimately causing the back muscles to lose their roles as stabilizers.

The carriage back angle is involved in the range of motion required to pull the knee joint anteriorly to the hip joint. There was a positive correlation between this angle and activation of the IO and RA muscles, and the experienced subjects showed greater ranges of activation. To perform this exercise accurately, strong activation of the abdominal muscles is required. In order to draw out strong activation of the IO muscles, it is necessary to move the carriage back to the stopper of the reformer, which is the starting point. The experienced subjects also had a larger range of motion in their knee stretch angles when performing the task. The knee stretch angle was negatively correlated with IO activation. A larger knee stretch angle requires stronger activation of the local muscles, indicating that this exercise can be a favorable intervention strategy to strengthen the IO muscles.

Proximal stability is a clinically intriguing topic that plays an important role in postural balance during activities of daily living. It can decrease the risk of musculoskeletal injury and is an imperative factor in rehabilitation. When integrating exercises to reinforce proximal stabilization, it is necessary to facilitate the activation of local muscles over global muscles; thus, Pilates can be an efficient method for this. Limitation of this study was that the flexibility and the strength levels of the participants could have influenced the performance of the exercises. Further research investigating the kinetics and kinematics of Pilates is needed to establish the use of Pilates in the field of rehabilitation.

## 5. Conclusions

In this study, muscle activation and quality of movement of the abdominal and lumbar muscles during Pilates-based stabilization exercise were analyzed to investigate the interaction of segments that control movement to provide stability and the segments that provide mobility for performance. The results of this study showed that subjects experienced in Pilates-based stabilization exercise efficiently activated the local abdominal and back muscles to stabilize the pelvis and trunk. In addition, the experienced subjects moved in a larger range of motion in the correct manner for the movement. Therefore, Pilates can be an effective way to activate local muscles in order to provide proximal stability.

## Figures and Tables

**Table 1:** General characteristics of subjects.

Variables	Experienced (*n* = 16)	Non-Experienced (*n* = 16)	*t*	*p*
Age (years)	26.75 ± 8.87	25.88 ± 4.85	0.346	0.732
Height (cm)	162.75 ± 5.09	162.63 ± 4.93	0.070	0.944
Weight (kg)	52.69 ± 5.73	53.19 ± 6.26	−0.235	0.815
Body mass index (kg/m^2^)	19.83 ± 1.18	20.06 ± 1.06	−0.460	0.648

Values are expressed as mean ± standard deviation. *p* value < 0.05 was considered statistically significant.

**Table 2:** Muscle activity during the knee stretch exercise (μV).

Variables		EAP (A)	RPP (B)	FNP (C)	Group	Position	Interaction
					*F*(*p*)	*F*(*p*)	*F*(*p*)
IO	Experienced	26.77 ± 19.73	33.35 ± 26.14	27.87 ± 22.27	12.261	0.515	2.272
	Non-experienced	12.71 ± 5.30	9.84 ± 2.46	11.06 ± 4.15	(0.000)	(0.600) B|AC	(0.112)
RA	Experienced	2.88 ± 1.69	3.41 ± 3.73	3.59 ± 3.16	0.404	0.386	0.870
	Non-experienced	2.89 ± 1.28	3.04 ± 2.19	2.57 ± 1.40	(0.530)	(0.681)	(0.424)
IO/RA	Experienced	9.10 ± 2.90	12.25 ± 6.78	8.43 ± 3.20	21.604	1.385	4.804
	Non-experienced	5.25 ± 3.73	4.40 ± 2.39	5.61 ± 3.84	(0.000)	(0.258)	(0.012)
MU	Experienced	9.62 ± 7.07	7.65 ± 6.12	8.51 ± 3.75	0.887	1.112	2.226
	Non-experienced	6.95 ± 4.92	7.40 ± 5.22	6.54 ± 3.56	(0.354)	(0.335)	(0.117)
IL	Experienced	9.23 ± 5.73	5.16 ± 3.96	6.78 ± 2.73	1.465	32.123	2.164
	Non-experienced	10.97 ± 7.05	6.42 ± 4.27	10.16 ± 6.80	(0.236)	(0.000)	(0.133)
						A|BC	
MU/IL	Experienced	1.06 ± 0.54	1.72 ± 0.99	1.42 ± 0.89	7.967	15.176	4.102
	Non-experienced	0.64 ± 0.15	1.19 ± 0.41	0.73 ± 0.27	(0.009)	(0.000)	(0.027)
						B|AC	

Values are expressed as mean ± standard deviation. IO, internal oblique; RA, rectus abdominis; MU, multifidus; IL, iliocostalis lumborum; EAP, extended back with anterior tilted pelvis; RPP, round back with posterior tilted pelvis; FNP, flat back with neutral pelvis.

**Table 3:** Kinematic analysis during the knee stretch exercise.

Variables		EAP (A)			RPP (B)			FNP (C)			Group	Position	Interaction
											*F*(*p*)	*F*(*p*)	*F*(*p*)
Pelvic stability	Experienced	94.50	±	56.46	64.88	±	35.78	61.13	±	26.34	8.741	6.772	1.349
(number)	Non-experienced	130.00	±	60.24	90.50	±	34.72	117.50	±	65.38	(0.006)	(0.002)	(0.267)
												A|B|C	
Trunk stability	Experienced	7.80	±	1.80	9.48	±	2.64	7.42	±	1.62	7.698	4.119	1.560
(angle)	Non-experienced	9.33	±	2.39	13.81	±	7.50	12.56	±	9.09	(0.009)	(0.021)	(0.219)
												B|AC	
Carriage back angle	Experienced	13.98	±	6.18	19.32	±	4.95	14.18	±	6.80	6.444	37.187	0.141
(degree)	Non-experienced	8.33	±	7.02	14.42	±	7.01	8.66	±	6.48	(0.017)	(0.000)	(0.869)
												B|AC	
Knee stretch angle	Experienced	31.91	±	4.60	28.70	±	4.87	33.95	±	5.10	8.848	12.947	5.173
(angle)	Non-experienced	26.90	±	5.60	25.59	±	5.79	26.71	±	5.74	(0.006)	(0.000)	(0.008)
												B|C	

Values are expressed as mean ± standard deviation. EAP, extended back with anterior tilted pelvis; RPP, round back with posterior tilted pelvis; FNP, flat back with neutral pelvis.

**Table 4:** Correlation between muscle activation and kinematic data.

	Pelvic Stability	Trunk Stability	Carriage Back Angle	Knee Stretch Angle
IO	−0.145	−0.064	0.444 *	0.506 *
RA	−0.188	−0.302 *	0.518 *	0.274 *
IO/RA	−0.419 *	−0.222 *	0.167	0.281 *
MU	−0.244 *	−0.142	0.133	0.027
IL	−0.299 *	−0.010	0.174	0.090
MU/IL	−0.134	−0.081	0.055	0.241 *

IO, internal oblique; RA, rectus abdominis; MU, multifidus; IL, iliocostalis lumborum. * *p* < 0.05.

## Data Availability

Not applicable.
